# Social Support Across Adversity Subtypes: A Latent Profile Analysis of Depression and Suicidal Ideation in College Students

**DOI:** 10.3390/bs16101934

**Published:** 2026-10-17

**Authors:** Wenshuang Long, Yuting Wang, Tian Zhou, Qiang Bao

**Affiliations:** 1 Mental Health Education Service Center, Southwest University, Chongqing 400715, China; 2 Faculty of Psychology, Southwest University, Chongqing 400715, China; wangyut@email.swu.edu.cn (Y.W.); zt2693689898@email.swu.edu.cn (T.Z.)

**Keywords:** indirect associations, social support, adversity subtypes, depression, suicidal ideation, college students, latent profile analysis

## Abstract

According to the theory of ecological systems and cumulative risk, this study used latent profile analysis to classify college students into adversity subgroups and examined the indirect associations of subgroup membership with depression and suicidal ideation through parental and teacher support. A total of 1142 college students were involved in the cross-sectional study. Three adversity profiles were identified: a multiple-adversity high-risk group (6%), a parental-control risk group (28%), and a low-risk group (66%). Depression and suicidal ideation were higher with increasing adversity risk. Parallel mediation analysis showed that the subtypes of adversity were independently associated with depression via parental and teacher support in all high-risk groups. Only the indirect route via teacher support reached the corrected significant threshold for both risk groups of suicidal ideation, and the parental support path did not reach this level in either risk group. As the multiple-adversity high-risk group comprised only about 6% of the sample, its estimate is relatively inaccurate and should be used with caution. This cross-sectional study identified patterns of adversity and associations of parental and teacher support with depression and suicidal ideation but cannot establish causality or protection.

## 1. Introduction

College students are at a critical stage of development between adolescence and adulthood, and the accumulation of academic, social and family problems during this period has made them a high-risk group for depression and suicidal ideation (Yao et al., 2024; Xiao et al., 2024; Ge et al., 2024). As essential indicators of predicting extreme psychological behavior in young adults, depression and suicidal ideation harm both individuals’ physical and mental health and academic progress and are thus a significant problem for mental health management at universities (Fang et al., 2024).

Given the above serious consequences, it is necessary to learn about the source and spread of such misfortune in a college student’s life. According to the ecological systems theory, people’s mental health is affected by the interaction of multiple environments, such as family, school, and the Internet, and continuous exposure to adverse conditions at these many levels is a major reason for poor mental health (Bronfenbrenner & Morris, 2006). Most of the previous studies that have used this framework have considered only the family and school as the main sources of stress for college students. Most college students’ social life is now conducted online, and thus the ecological space for adversity has expanded beyond family and school into the digital realm. In this digital environment, cyberbullying has become a new and serious risk factor that is different from traditional family and school problems, and it can increase the damage caused by offline negative events. Most of the existing research is still in the form of a variable-centered model, and each type of adversity is treated as an independent source of risk; therefore, the homogeneity of students’ exposure to adversity is implicitly assumed, and the differentiated combinatorial patterns that students actually face in real life are not considered. The assumption of homogeneity fails to consider the different experiences of adversity among individuals and therefore cannot explain the varied psychological results shown by people in similar risk situations (Yu et al., 2023). Person-centered studies of emerging adults and college students have begun to address this problem by identifying subgroups with co-occurring childhood adversities, general life events, and financial or social risks, and by linking these subgroups to depression, suicidal ideation, and well-being (Navarro Flores & Berzenski, 2025; Newcomb-Anjo et al., 2017; J. Tang et al., 2023). None of the three studies included cyberbullying victimization in their categories. To our knowledge, no research has been conducted to combine family, school, peer, and online adversities into a single category and compare the association of parental and teacher support among these profiles.

Cumulative risk theory suggests that the harm to mental health resulting from adversity occurs in stages, and as the number of risk factors increases, so does the severity of psychological damage (Evans et al., 2013). Previous studies have found that accumulated adversity is associated with depression and suicidal ideation among college students (Yao et al., 2024; J. Zhang et al., 2022; Zhou et al., 2023; Park, 2025). However, two prominent deficiencies remain. Firstly, most previous research has focused on traditional offline harm; the separate and combined impact of cyberbullying is not covered, so it is unsuitable for the current ecological situation of college students. Secondly, they are still equally weighted additive indices that do not differentiate between the quantitative cumulative effects and the qualitative configurational differences in adversity and are thus limited in explanatory power. Latent profile analysis (LPA) can be employed in a person-centered way to classify adversity subtypes according to individuals’ actual exposure patterns, precisely delineate heterogeneous risk structures, and thereby address the deficiencies of the conventional variable-centered approach. The present study therefore aims to identify the different features of adversity risk more accurately, to examine how social support is associated with outcomes across adversity profiles, and to provide theoretical and practical support for the construction of a personalized intervention system.

Social support is widely regarded as a primary protective factor that can mitigate the negative effects of adverse circumstances (Cohen & Wills, 1985; Song et al., 2026). For college students, the two most stable support sources are parental autonomy support and teacher autonomy support; they can help reduce the negative effects of familial and school-based adversity, respectively (Sun et al., 2023; Tan et al., 2024; Zeng et al., 2024; Chai & Shek, 2024). Existing research has focused primarily on the general protective effect of a single support area and has given insufficient attention to the ecological specificity and outcome differences in support associations. When the family simultaneously serves as the environment for individual growth and the origin of difficulties, parental support may be impaired in function; at the same time, teacher support operates outside the family system and can provide compensatory protection. To date, the field has not systematically studied how these two support forms are differently associated with outcomes in different adversity subtypes and across various psychological outcomes, leaving the conditions under which the association of parental support with better outcomes is weakened largely unexplored.

Based on a relatively large sample of Chinese college students, the present study uses the person-centered approach of latent profile analysis to systematically study the combination and distribution patterns of adversity risk in the family, school and online ecological environments. It also explores the connections among various types of adversity exposure and two mental health problems, depression and suicidal thoughts, and uses a parallel dual-mediation model to examine the indirect paths through parental support and teacher support for these relationships. As these data are cross-sectional, only paths of statistical association can be found; causality and time cannot be determined. Based on the above theoretical development and empirical evidence, the present study puts forward the following three hypotheses: (1) The exposure patterns of multi-ecological adversity among college students show significant individual differences and can be clustered into several distinct adversity subtypes, each with a different combination of risks in multiple areas. (2) Environmental adversity is associated with depression and suicidal ideation in a graded and cumulative manner; that is, different adversity subtypes correspond to different degrees of depression and suicidal ideation, and individuals who face a large number of adverse circumstances are likely to report higher levels of psychological distress due to the overlap of adverse configurations. (3) Parental support and teacher support are expected to show indirect associations linking adversity subtypes with depression and suicidal ideation. Because parental support originates from the family and teacher support originates from outside the family, these two indirect associations are expected to differ in strength across adversity subtypes, especially under high-risk conditions where many adversities occur simultaneously.

## 2. Methods

### 2.1. Participants

From November to December 2025, 1226 undergraduates from several universities in Southwest China were recruited by convenience sampling. The survey was carried out on the Wenjuanxing platform (Changsha Ranxing Information Technology Co., Ltd., Changsha, China; https://www.wjx.cn/) via mobile phone or computer. Before taking the questionnaire, the participants read the study information sheet and the informed consent form, which explained the purpose of the study, contents of the questionnaire, anonymization measures, use of data, voluntary participation and the right to withdraw at any time without reason; only after agreeing to these conditions did they proceed to complete the questionnaire. At the end of the questionnaire, all participants were provided with information on the university’s counseling services and a psychological crisis hotline; those who screened positive for high suicidal ideation were automatically shown a prompt to seek professional psychological help. Three exclusion criteria were used: straight-line responding, defined as selecting the same response option for at least 80% of items (*n* = 36); completion time shorter than one-third of the mean completion time of the sample (*n* = 27); and missing data for the main variables of depression or suicidal ideation (*n* = 21). According to the above exclusion criteria, the final valid sample is 1142 students, and the actual response rate is 93.15% (1142/1226). The three exclusion criteria did not overlap (36 + 27 + 21 = 84 = 1226 − 1142). The age range of the participants was 18–23 years old, and the mean age was 19.26 years (standard deviation, SD = 0.978); there were 587 men (51.40%) and 555 women (48.60%). This study was approved by the Institutional Review Board of Southwest University (approval code: SWU-ECHR-20250271; approval date: 10 November 2025). Participants provided informed consent to participate.

### 2.2. Measures

#### 2.2.1. Parental Psychological Control

The scales for parental psychological control in this study were developed by Shek et al. (2006) and Shek (2007), based on a systematic review of previous literature (Barber, 1996). This scale has been well validated among college students with satisfactory psychometric properties (Wu et al., 2026). The five main types of parental psychological control are: invalidating emotions, excessive regulation, withdrawal of affection, limitation of free speech, and personal criticism. The two parallel subscales of the scale are paternal and maternal psychological control, respectively, and each subscale has 10 identical items that differ only in their reference to fathers and mothers. All the items were rated on a 5-point Likert scale, and ‘1’ was “strongly disagree” and ‘5’ was “strongly agree”. The mean scores of the respective 10-item subscales for paternal and maternal psychological control were calculated separately, and these two subscale scores were used as separate variables rather than being combined into a single parental-psychological-control score. Higher mean scores indicated greater perceived paternal or maternal psychological control, respectively. The internal-consistency coefficients of Cronbach’s alpha for the paternal subscale and the maternal subscale were 0.78 and 0.80, respectively.

#### 2.2.2. Exposure to Domestic Violence

Exposure to domestic violence was assessed using the modified Conflict Tactics Scale (CTS), adapted from the Chinese-revised Conflict Tactics Scales validated by Tiwari et al. (2007). The three attributes of the original 16 items are bargaining, psychological aggression and physical aggression. All items were rated on a 6-point Likert scale, from 1 (never) to 6 (always). Given that the present study focused on the impact of parental violence exposure among college students, the bargaining dimension was excluded, and only the psychological aggression and physical aggression sub-dimensions were retained. The mean score of exposure to domestic violence was calculated across the items in the two retained subdimensions, with higher mean scores indicating more frequent exposure to domestic violence. The Cronbach’s α coefficient for the composite of the retained items in this study was 0.79.

#### 2.2.3. Deviant Peer Affiliation

Deviant peer affiliation was assessed by the eight-item Deviant Peer Affiliation Scale (Li et al., 2013, 2016). Although this scale was originally designed for adolescent samples, it has been validated and widely applied among Chinese college students (e.g., Jiang et al., 2026). Respondents were asked to rate the proportion of their friends engaged in particular deviant behaviors (e.g., alcohol consumption, shoplifting, skipping school) over the past year on a scale of 1 (none) to 5 (all). The mean score of the eight items was calculated, with higher mean scores indicating greater affiliation with deviant peers. The internal-consistency coefficient of the scale is Cronbach’s α = 0.84.

#### 2.2.4. School Bullying Victimization

Olweus’s Bullying Questionnaire, which was developed by Olweus (1993) and revised by W. X. Zhang et al. (1999), was used to assess the experiences of college students with school bullying (He et al., 2025). An example item is: “I was deliberately hurt by peers through nicknames or damage to my property”, and the corresponding responses were recorded on a 5-point Likert scale, with 1 meaning “never experienced” and 5 meaning “experienced every day”. The mean score of the items was calculated, with higher mean scores indicating more frequent school-bullying victimization. The internal-consistency coefficient of Cronbach’s α for the scale is 0.88.

#### 2.2.5. Cyberbullying Victimization

The Chinese version of the Cyberbullying Inventory for College Students (CICS-CV), originally developed by Francisco et al. (2015) and revised by L. L. Tang et al. (2022), was used in this study to evaluate the experiences of college students of cyberbullying victimization in the past six months. The nine items of the scale are on a 5-point Likert scale, and “never” is 1 and “several times a week” is 5. The mean score of the nine items was calculated, with higher mean scores indicating more frequent cyberbullying victimization. It has a reliability coefficient of Cronbach’s α = 0.82.

#### 2.2.6. Depression

The nine components of the Patient Health Questionnaire-9 (PHQ-9) are used to assess depressive symptoms (Kroenke et al., 2001), including anhedonia, sad mood, sleep, energy, appetite, guilt, concentration, motor function, and thoughts of death or self-harm. The range for the rating of the original items is 1 (never) to 4 (almost every day). The mean score of the nine items was calculated, with higher mean scores indicating more severe depressive symptoms. Depression was analyzed as a continuous variable, and no cutoff score was applied. It has reasonable reliability and internal consistency (0.84).

#### 2.2.7. Suicidal Ideation

Suicidal ideation was assessed using the Adolescent Suicidal Ideation Scale, a Chinese adaptation of the Positive and Negative Suicide Ideation (PANSI) Inventory developed by Osman et al. (1998), which was further adapted for Chinese high school students by X. Z. Wang et al. (2011). This scale has been widely adopted and validated for use among Chinese college students in recent empirical studies (e.g., Huang et al., 2026; Wu et al., 2026). There are 14 items in total for the two types: positive suicidal ideation and negative suicidal ideation. All the items were rated on a 5-point Likert scale, and ‘1’ was ‘never’ and ‘5’ was ‘always’. Items in the positive suicidal ideation dimension were reverse-scored specifically. After the positive suicidal ideation items were reverse-scored, the mean score of all 14 items was calculated, with higher mean scores indicating greater suicidal ideation. The Cronbach’s α for the total scale was 0.81.

#### 2.2.8. Parental Support

Parental autonomy support was measured by the 12-item scale adapted from Q. Wang et al. (2007), which assesses the extent to which parents support the independence of children living at home. All items were rated on a 5-point Likert scale, and “strongly disagree” was 1 and “strongly agree” was 5. The mean score of the items was calculated, with higher mean scores indicating greater perceived parental autonomy support. Cronbach’s alpha of the internal-consistency coefficient for the scale is 0.83.

#### 2.2.9. Teacher Support

Teacher support is shown in the revised Chinese edition of the Learning Climate Questionnaire (LCQ). The scale was first developed by Williams and Deci (1996), and later J. Wang et al. (2017) adapted it for use with Chinese students to investigate students’ general perceptions of teacher autonomy support in school. The revised scale has fourteen items. All the items were given a 5-point Likert scale, and “strongly disagree” was 1 and “strongly agree” was 5. The mean score of the items was calculated, with higher mean scores indicating greater perceived teacher autonomy support. The internal-consistency reliability coefficient of the scale is 0.87.

### 2.3. Statistical Analysis

Analysis was conducted in R version 4.5.3 (R Foundation for Statistical Computing, Vienna, Austria). Scale scores were calculated as the mean of all items after reverse-coding the relevant items. As there were no missing data in the analytic variables, a complete-case analysis was used (cases with missing data on the main variables had already been excluded; see Section 2.1). We standardized (z-scored) the six adversity indicators prior to latent profile analysis (LPA). One-through-six-profile solutions were estimated with Model 1 in tidyLPA version 1.1.0, which assumes equal indicator variances across profiles and zero within-profile covariances. Akaike Information Criterion (AIC), Bayesian Information Criterion (BIC), sample-size-adjusted BIC (aBIC), entropy, profile size, posterior probabilities, parsimony and interpretability were used in model selection.

A retained solution was selected, and then separate analyses of covariance (ANCOVAs) were conducted, with sex and age as covariates, to examine the differences in PHQ-9 depressive symptoms and suicidal ideation. Sex and age were included as covariates. Significant omnibus tests were followed by Tukey’s honestly significant difference (HSD) test. Partial eta squared (η^2^_p_) is the effect size, and covariate-adjusted means and 95% confidence intervals (CIs) are presented.

Then, parallel indirect-association models were estimated using the low-risk group as the reference group, so that the three-category profile predictor was represented by two contrasts (Cao et al., 2023), and parental and teacher support were added simultaneously as mediators. Indirect associations were obtained from 2000 bootstrap replicates. For each outcome, the four pre-determined indirect associations resulting from the two profile contrasts and the two mediators formed a single multiplicity family. To control the family-wise Type I error rate at 0.05, a Bonferroni-adjusted α of 0.0125 (0.05/4) was employed. Therefore, the inference of indirect associations is based on the 98.75th percentile bootstrap confidence interval. We report the direct and total path estimates and their 95% confidence intervals.

Two sensitivity analyses were conducted to verify the results. First, PHQ-9 Item 9 assesses thoughts of death or self-harm, which are conceptually related to suicidal ideation. Therefore, we used the eight-item Patient Health Questionnaire (PHQ-8) to calculate the depression score and excluded Item 9. Then, ANCOVA and indirect-association analyses were carried out using PHQ-8 as the outcome. Second, to investigate the stability of the profile enumeration, the LPA was re-estimated with an alternative two-profile solution, which provides the most parsimonious comparison with the three-profile model. Covariate-adjusted outcome comparisons and parallel indirect-association models were performed on the resulting two-profile classification.

## 3. Results

### 3.1. Descriptive Statistics and Bivariate Correlations

All adversity indicators were positively correlated with depression and suicidal ideation, whereas parental and teacher support were negatively correlated with both outcomes (all *ps* < 0.001; Table 1).

### 3.2. Latent Profile Analysis

One to six profiles of latent profile models were estimated using the six adversity indicators (Table 2). The three-profile solution of the model improved the fit considerably. The three-profile solution had a high classification precision (entropy = 0.92), and the bootstrap likelihood-ratio test (BLRT) showed that it was significantly better fitted than the two-profile solution. However, the addition of a fourth profile did not improve the model fit. Although some information criteria continued to decline with an increase in the number of profiles, the four-, five- and six-profile solutions had empty or negligible profiles and showed lower entropy values; thus, their classification performance was worse. Therefore, the three-profile solution was selected due to its good combination of statistical fit, classification accuracy, profile size, parsimony and substantive interpretability.

**Figure 1:**
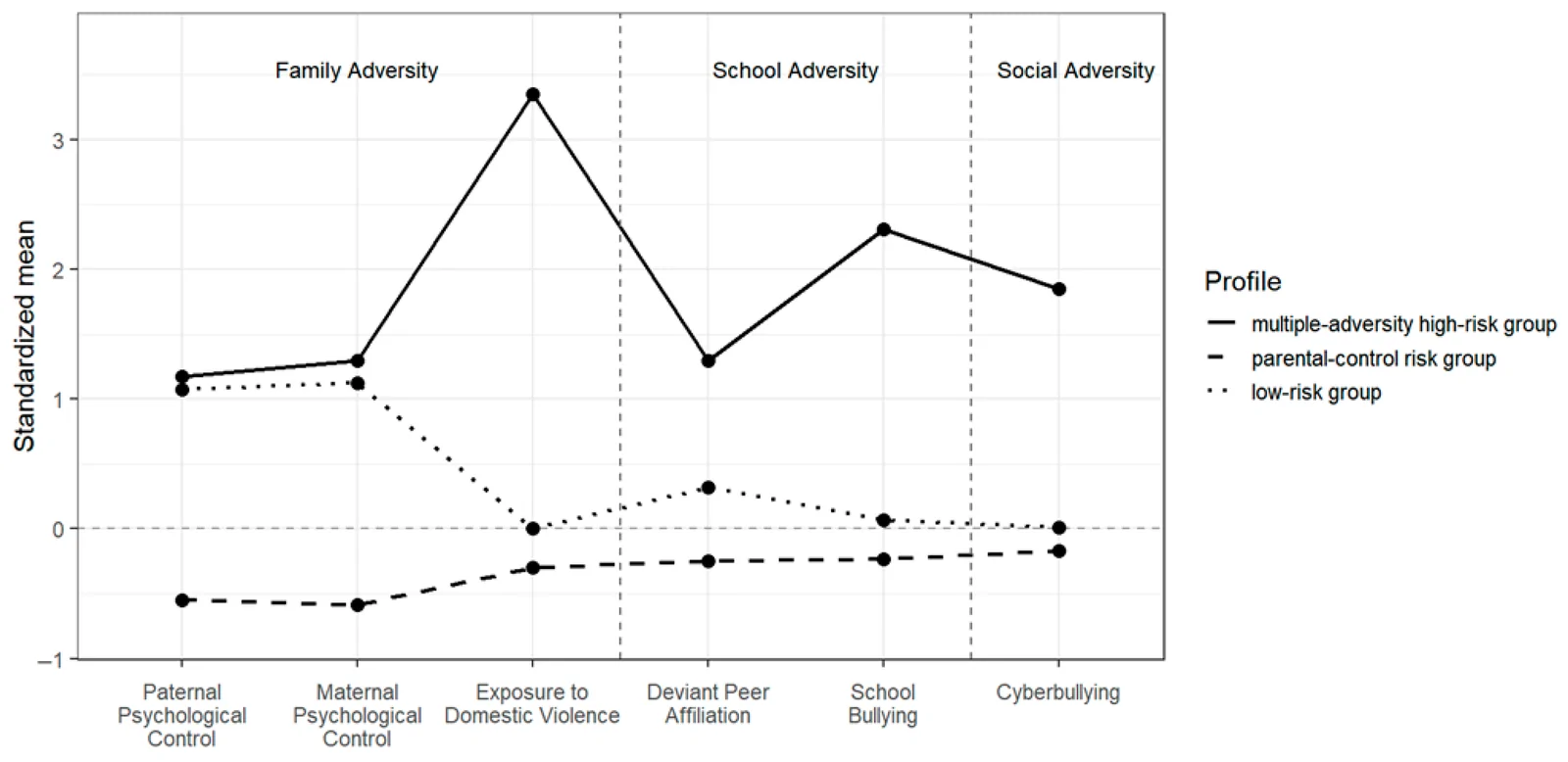
Latent profile plot for adversity indicators (*N* = 1142). Note. Class 1 = multiple-adversity high-risk group (*n* = 68); Class 2 = parental-control risk group (*n* = 316); Class 3 = low-risk group (*n* = 758).

Figure 1 shows the three retained latent profiles. Class 1 was set up as a multiple-adversity high-risk group, accounted for 6% of the analytical sample, showed higher standardized scores in all six adversity indicators, and was particularly vulnerable to school bullying and cyberbullying victimization and domestic violence. Class 2 was set up as the parental-control risk group, made up 28 percent of the total, and was mainly characterized by high levels of parental-control anxiety for both parents; peer-, school-, and cyber-related adversity was relatively low. Class 3 was designated as the low-risk group and accounted for 66% of the sample, and it had low scores in all six adversity indicators. Therefore, the three-profile solution divided the small multi-context adversity group, the larger family-control-specific risk group, and the majority low-risk group. The mean posterior probability of membership in the assigned profile for each class was high: 0.990 for Class 1, 0.941 for Class 2, and 0.974 for Class 3. The complete matrix of average posterior class-membership probabilities is provided in the Supplementary Table S1.

### 3.3. Profile Differences in Depression and Suicidal Ideation

ANCOVA was used to adjust for sex and age. Profile membership was significantly associated with depression, F(2, 1137) = 96.68, *p* < 0.001, η^2^_p_ = 0.145. The adjusted mean depression scores were 2.256 (95% CI [2.132, 2.380]) for the multiple-adversity high-risk group, 1.692 (95% CI [1.634, 1.749]) for the parental-control risk group, and 1.434 (95% CI [1.397, 1.471]) for the low-risk group. All Tukey-corrected pairwise comparisons were statistically significant (all *p* < 0.001).

**Figure 2:**
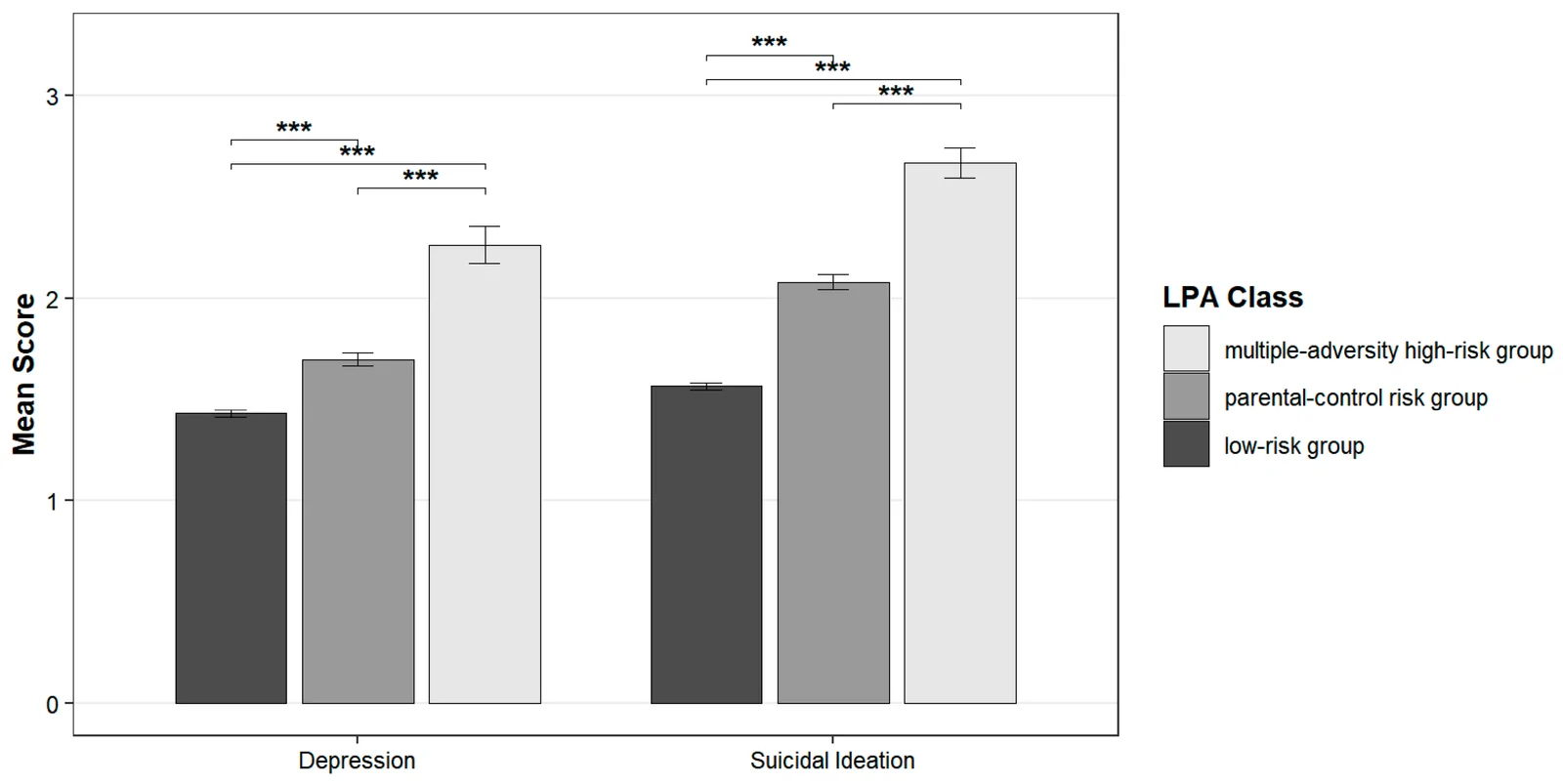
Covariate-adjusted profile differences in depression and suicidal ideation. Note. *** *p* < 0.001, means are adjusted for sex and age.

Profile membership was also significantly associated with suicidal ideation, F(2, 1137) = 198.14, *p* < 0.001, η^2^_p_ = 0.258. The adjusted mean scores of suicidal ideation for the multiple-adversity high-risk group, parental-control risk group, and low-risk group were 2.662 (95% CI [2.532, 2.792]), 2.076 (95% CI [2.016, 2.136]) and 1.567 (95% CI [1.528, 1.605]), respectively. All Tukey-corrected pairwise comparisons were statistically significant (all *p* < 0.001). Scores for both depression and suicidal ideation were highest in the multiple-adversity high-risk group, followed by the parental-control risk group and the low-risk group (Figure 2). Complete covariate-adjusted omnibus tests, adjusted means and confidence intervals are presented in Supplementary Table S2.

### 3.4. Parallel Mediation Models

Parallel indirect-association models were employed, and the low-risk profile was taken as the reference group. Therefore, the profile presented in the above figure is selected as the reference because it has the largest proportion of the sample (66.37%) and a lower overall level of adversity; thus, it provides a theoretically sound and statistically stable basis for comparison with other risk profiles. Therefore, the estimated coefficients are the differences in parental and teacher support and the outcomes for each risk group compared to the low-risk group. Parental and teachers’ support were also added as mediators. Four pre-defined indirect associations for each outcome, consisting of two profile contrasts (Class 1 vs. Class 3 and Class 2 vs. Class 3) through the two mediators, a single multiplicity family was formed. To control the family-wise Type I error rate at 0.05, a Bonferroni-adjusted α of 0.0125 was used (Lee & Lee, 2018). Therefore, the inference for the indirect associations was based on the 98.75th percentile bootstrap confidence interval, and the direct and total path estimates were shown with 95% confidence intervals. The results of the PHQ-8 sensitivity analysis model are presented in Supplementary Table S4.

### 3.5. Indirect Associations for Depression

**Figure 3:**
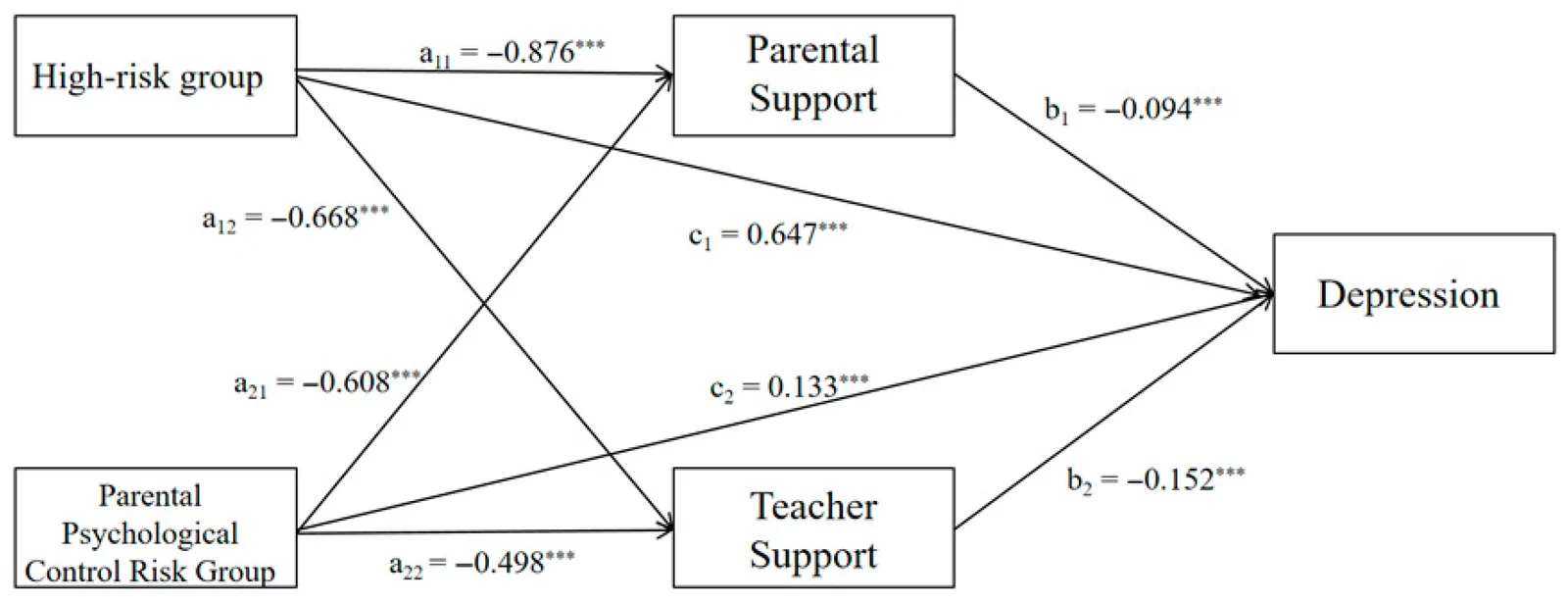
Parallel mediation model for depression. Note. The low-risk group was the reference category. *** *p* < 0.001.

As shown in Table 3 and Figure 3, the risk profile contrast also had a higher total and direct risk of depression than the low-risk group. For the multiple-adversity high-risk contrast, indirect effects were found via parental support, B = 0.082, 98.75% CI [0.026, 0.154], and teacher support, B = 0.102, 98.75% CI [0.057, 0.159]. For the parental-control risk contrast, indirect effects were also found via parental support, B = 0.057, 98.75% CI [0.019, 0.103], and teacher support, B = 0.076, 98.75% CI [0.044, 0.109]. Therefore, both support paths reached the corrected significance level for depression, and the direct profile differences were still statistically significant.

### 3.6. Indirect Associations for Suicidal Ideation

**Figure 4:**
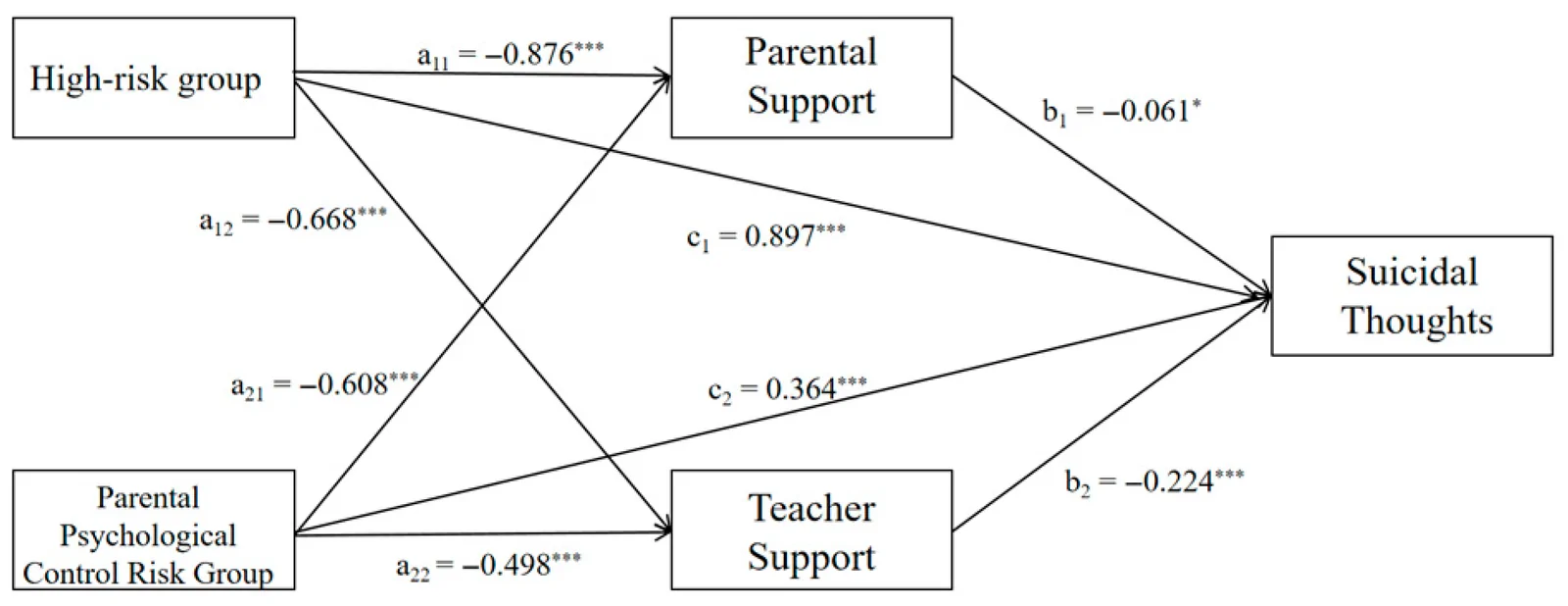
Parallel mediation model for suicidal ideation. Note. The low-risk group was the reference category. * *p* < 0.05, *** *p* < 0.001.

Both risk-profile comparisons showed an increase in the direct and total associations with suicidal ideation compared to the low-risk group (Table 4 and Figure 4). For the multiple-adversity high-risk contrast, the indirect effect via teacher support was significant, B = 0.150, 98.75% bootstrap CI [0.089, 0.224], and the indirect effect through parental support was B = 0.054, 98.75% bootstrap CI [−0.011, 0.127]. For the parental-control risk contrast, the indirect effect through teacher support was also significant, B = 0.112, 98.75% bootstrap CI [0.069, 0.157], and the indirect effect through parental support was B = 0.037, 98.75% bootstrap CI [−0.007, 0.082]. Therefore, teacher-support indirect associations were supported in both risk-profile comparisons; however, neither parental-support indirect association met the multiplicity-adjusted criterion.

### 3.7. Sensitivity Analyses

PHQ-8 scores were positively correlated with suicidal ideation (r = 0.516, 95% CI [0.472, 0.557]). After adjustment for sex and age, the PHQ-8 scores of the three latent profiles were different, F(2, 1137) = 84.20, *p* < 0.001, η^2^_p_ = 0.129. The pattern of the specific indirect association was the same as that of PHQ-9; the 98.75% bootstrap confidence intervals for the indirect associations via parental and teacher support both excluded zero in the risk-profile contrast. Detailed results of the PHQ-8 sensitivity analysis are shown in Supplementary Tables S2–S4.

To investigate the stability of profile enumeration, we employed the same six adversity indicators to re-estimate the LPA and specified a two-profile solution. This solution had a lower risk profile (*n* = 809, 70.8%) and a higher risk profile (*n* = 333, 29.2%) (Supplementary Table S5). The Classification Accuracy was relatively high (Entropy = 0.886; Mean Assigned Posterior Probabilities = 0.977 and 0.945). After adjusting for sex and age, the high-risk group had higher scores in PHQ-9, suicidal ideation and PHQ-8 than the low-risk group (all *p* < 0.001; Supplementary Table S6). In the two-profile model, the 98.75% bootstrap confidence intervals for the indirect associations through teacher support excluded zero for all three outcomes. The corresponding indirect associations via parental support did not include zero in the confidence intervals for PHQ-9 and PHQ-8 but did for suicidal ideation (B = 0.051, 98.75% bootstrap CI [−0.0004, 0.103]). Direct and total associations between profile membership and all three outcomes were also statistically significant (all *p* < 0.001; Supplementary Table S7).

## 4. Discussion

The causes of depression and suicidal thoughts among college students are varied, and their manifestations are also inconsistent. Drawing on ecological systems theory and cumulative risk theory (Evans et al., 2013), this study employed latent profile analysis to explore the different forms of adversity in family, school and online environments. Three types were found: a large number of students in a low-risk group, a parental-control risk group, and a small multiple-adversity group with concurrent exposure in multiple ecological domains. Depression and suicidal ideation were also higher across these profiles. Both parental support and teacher support showed indirect associations with depression in the higher-risk groups. However, the two ways of leading to suicidal ideation were different: the indirect path through parental support did not reach the corrected significance level in either risk group, whereas the indirect path through teacher support remained significant in both. Adversity is positively correlated with both outcomes, and after controlling for the two support paths, it is directly associated with them. As a cross-sectional study, it can only show associations among the variables and cannot determine their cause or prevention. This study can provide support for future research on stratified prevention and targeted intervention of depression and suicidal ideation in this group (Sun et al., 2023; Yu et al., 2023).

### 4.1. Adversity Subtypes and Cumulative Risk Patterns Among College Students

Latent profile analysis of parental psychological control, family violence exposure, deviant peer affiliation, school bullying, and cyberbullying victimization yielded three subtypes: a multiple-adversity group (6%), a parental-control risk group (28%), and a low-risk group (66%). Sun et al. (2023) extended the previous LPA research on early adolescents to Chinese college students by adding cyberbullying to the list of family and school problems. Most students (over 60%) are in a relatively low-risk group, and almost a third (about 30%) face only a family problem of parental control without other school or online risks. Only a small number are under the combined pressure of family, school and online life and are thus in a vulnerable position. Adversity exposure is divided into groups, and therefore, the risk-homogeneity assumption of traditional variable-centered research is violated.

In terms of how adversity relates to risk, the current findings show that multi-ecological adversity is associated with higher levels of depression and suicidal ideation among college students. The three adversity profiles were associated with depression and suicidal ideation in a graded pattern in the current sample, with the multiple-adversity group having the highest scores, followed by the parental-control-risk group, and the low-risk group having the lowest. According to the basic premise of cumulative risk theory, the spread of risk across various domains may be more damaging to mental health than a single large risk event (Evans et al., 2013). Here, the graded pattern was recognized as one of the features of the qualitatively different structures found by LPA; thus, cumulative risk theory and person-centered classification can be regarded as complementary systems, and LPA has revealed the underlying structure of the gradient.

Both outcomes showed the same graded pattern in the three subtypes; therefore, overlapping multi-ecological adversity was associated with higher depression and suicidal ideation, which aligns with the social ecological model of suicide and self-injury in adolescents (Ban et al., 2025). As the two outcomes were measured on different scales, the current data can only support a comparison of the pattern of associations, not the magnitude of associations across the outcomes. At the same time, the parental-control risk group did not have additional school or online adversity and was still more depressed and suicidal than the low-risk group; therefore, these findings suggest that parental psychological control is a family-based risk factor associated with poorer mental health even in the absence of other adversities (Beliveau et al., 2023; Bradshaw et al., 2025).

### 4.2. Differing Associations of Parental Support and Teacher Support Across Adversity Subtypes

Environmental protective resources are generally considered to be buffers against harm in times of adversity, but the value of different support resources will vary depending on the circumstances and the outcome (Cohen & Wills, 1985). In terms of risk-protection integration, this study has found that the indirect associations through parental support and teacher support are not uniform but vary according to the configuration of adversity and the outcome being investigated; thus, it addresses the limitation of previous research that focused on only one support source within a single risk scenario. As only indirect paths through support have been explored in these analyses, and the association between adversity and outcomes has not been tested for moderation, these are statistical pathways rather than buffering effects in a strict sense.

Support resources were associated with depression in both cases. Whether college students belonged to the single-domain parental-control risk pattern or the comprehensive high-risk pattern, including all domains, both parental support and teacher support showed an indirect association between adversity subtype and depression. Therefore, although both family and school support are linked to depression in the presence of single and multiple adversities, these cross-sectional data cannot determine whether such support is related to lower depression over time.

The indirect associations of suicidal ideation varied by adversity subtype. Under the single-domain parental-control risk configuration, where the family ecological system is likely to remain relatively unchanged, only the teacher support pathway met the corrected significance threshold; the parental-support pathway did not, and teacher support has been reported to moderate the links of family-related risk with adolescent suicidal ideation and peer interactions (Xu et al., 2025; Chen et al., 2024). Under the overall high-risk configuration, however, in cases where the family is the main source of problems and the parent–child attachment is damaged, the indirect association through parental support did not reach the corrected significance threshold; on the other hand, the indirect association through teacher support remained significant. Another possible explanation is that, in line with the resilience compensation model (Garmezy, 1985; Masten, 2001), when a family’s internal support system is weakened by multiple adverse events, the school ecology’s support for a child may still be associated with lower suicidal ideation, a pattern also consistent with attachment theory (Bowlby, 1969/1982). Longitudinal or experimental studies are needed to verify this interpretation, and it should not be assumed that parental support is absent or that teacher support alone is sufficient. A cross-sectional design cannot distinguish between a genuine decline in parental support and reverse causation or common-method bias, and due to a small multiple-adversity group, the power to detect this indirect association was limited. Bonferroni correction was also used for the four indirect associations; therefore, even small differences in the uncorrected *p*-values compared to the corrected threshold of *p* < 0.0125 should not be overestimated. Negative cognitive patterns, social withdrawal and a negative help-seeking experience that may occur under high-risk adversity can further weaken the link between parental support and better outcomes (Uno et al., 2024; Stewart et al., 2024).

### 4.3. An Integrated Risk-Protection Perspective

By integrating multi-ecological adversity risk with internal and external protective resources, the present study provides an integrated perspective on how different types of adversity, social support, depression and suicidal ideation are related, as well as the boundary conditions for family-based and school-based support: for suicidal ideation, the parental support pathway did not reach the corrected significance level in either risk group, but the teacher support pathway was supported in both risk groups and for both outcomes. These findings are preliminary and require replication. The proportion of support pathways in the association between adversity and the outcome was relatively small. In the multiple-adversity contrast, the direct associations (β = 0.273 for depression and β = 0.338 for suicidal ideation) were roughly three to four times larger than the sum of the two indirect associations (β = 0.078 and β = 0.076, respectively), and both direct associations remained significant in the risk contrasts. Therefore, most of the association between multiple adversities and the outcomes was not explained by parental or teacher support, and mediators such as peer support and individual resilience may account for part of the remaining direct associations.

At the same time, in this study, parental psychological control and parental support were treated as independent variables, and they were not in opposition but instead showed a moderate negative correlation (r = −0.31 for paternal and r = −0.39 for maternal psychological control); this result is consistent with the conclusions of prior meta-analyses and empirical research (Bradshaw et al., 2025). Families with high control can still provide some emotional support, but the association between this support and lower suicidal ideation under multiple adverse circumstances was not consistent; thus, this pattern is in line with research on social support and suicidal ideation (Kalgren & Brausch, 2025). The cultural background may also affect the interpretation of these results, and previous cross-cultural studies have found both similarities and differences. In a longitudinal study of seventh-grade students in the United States and China, parents’ psychological control predicted worse emotional function in both countries, and the benefits of parental psychological autonomy support were generally stronger in the United States than in China (Q. Wang et al., 2007). It has also been proposed that Western labels such as authoritarian parenting do not fully reflect Chinese child-rearing, and instead, the idea of training is used (Chao, 1994). Based on the above analysis, the higher depression and suicidal ideation linked to parental control found in this study may be broadly comparable across cultures; however, the non-significant parental-support pathway for suicidal ideation under adversity and the more consistent teacher-support pathway may partly be due to characteristics of the Chinese context, such as parenting practices and a collectivist campus culture that affect the perception of parental and teacher support. The current data cannot test this account; cross-cultural comparison of the same indicators is needed.

### 4.4. Limitations and Future Directions

Some deficiencies of the current study need to be acknowledged and corrected in future research. This study used a cross-sectional design with latent profile analysis to examine the associations and indirect paths among multi-ecological adversity, social support, depression and suicidal ideation; however, due to the limitations of this method, causality could not be determined, nor could changes in these factors over time be observed, and a bidirectional or reciprocal relationship among adversity exposure, perceived social support, and psychological outcomes remained uncertain. The pattern of parental support in the multiple-adversity group is a typical case: the design cannot distinguish between a genuine loss of protective function and reverse causation, such as when students with high suicidal ideation perceive or report less parental support, and neither account can be separated from common method variance because adversity, support and outcomes were all self-reported at the same time. Longitudinal designs, such as cross-lagged panel or longitudinal mediation models, and experimental or intervention studies need to be conducted before any causal or protective interpretation of these pathways can be made.

The current study has focused solely on parental support and teacher support as protective resources, excluding other potentially influential protective factors such as peer support, school climate or individual psychological resilience (Zou et al., 2022; Yan & Li, 2025); therefore, the scope of the risk-protection model is relatively narrow and some direct associations may not have been captured; future research could be enhanced by adding a wider range of ecological protective resources to fully explore the risk-protection dynamics in the whole ecological system. All the variables in this study were collected through self-report by college students; thus, subjective reporting bias and common method variance could not be avoided, and social desirability bias may have occurred, resulting in an underestimation of risk exposure and an overestimation of perceived support. In the future, multi-informant assessments and objective behavioral indicators can be added to improve the reliability of the results, as well as cross-validation across different measurement methods.

Measurement invariance of the instruments across gender was not examined. Therefore, it cannot be ruled out that the scale functions differently across gender groups, and we do not know whether the adversity profiles and indirect associations are the same across groups. In the future, measurement invariance tests and comparisons of profile solutions will be carried out across these groups. The proportion of the multi-adversity group in the sample was only about 6%, and therefore, based on this small number of participants, their adjusted means, pairwise comparisons and indirect associations could not be reliably determined. Bonferroni correction reduces the power for this subgroup even further, and thus, the non-significant parental-support result for suicidal ideation, particularly in this group, should be considered tentative. To verify the accuracy of this profile and related patterns, replication with a larger number of participants in the high-risk group is needed for stable estimation.

Convenience sampling was used to obtain the sample from universities in Southwest China, and online questionnaires were distributed; thus, it is not a probability sample. Therefore, the proportions of the three adversity profiles (6%, 28%, and 66%) only refer to this sample and should not be considered the actual proportion in the entire population; in addition, the structure of these profiles may vary by area and organization. The participants were also concentrated in a narrow age range, with a mean age of 19.26 years, so the results may not be applicable to older students, postgraduate students or young people outside of university, and students who chose to complete the online survey may be different from those who did not. Cultural context also limits generalizability. Only Chinese college students were selected as the sample, and the cultural contexts of China and the West differ significantly in terms of parenting styles, parent–child relationship dynamics, school-based developmental approaches, and other sociocultural norms. Authoritarian parenting and a collectivist campus climate may shape the experience of adversity and the operation of social support differently; thus, it is difficult to generalize the present findings on cumulative risk patterns and support-related associations directly to student populations in other countries or cultures. In the future, collect probability samples from multiple areas, expand the range of ages, and include various countries and cultures to verify the external validity of the current results and build an all-encompassing theoretical system for studying the connections among various ecological adversities and depression and suicidal ideation in different cultures. The current study has also focused primarily on the differences in psychological outcomes among empirically identified subtypes of adversity and has not examined the antecedent mechanisms that lead to these heterogeneous configurations; in the future, predictive variables such as individual traits, family function, and school environment can be added to further explore the developmental logic underlying the heterogeneity of adversity risk among college students (Yu et al., 2023).

## 5. Conclusions

Based on ecological systems theory, cumulative risk theory and attachment theory, this paper used latent profile analysis along with a parallel dual mediation model to explore the subtype clustering characteristics of multi-ecological adversity among college students and the cumulative risk pattern of their association with depression and suicidal ideation, as well as the different associations of parental support and teacher support with these outcomes from a risk-protection integration perspective. Three principal conclusions are drawn from the cross-sectional associations.

Subtypes of multi-ecological adversity exposure among college students and a graded cumulative risk pattern. The three categories of adversity in the family, school, and online environments were: an all-encompassing high-risk group, a parental-control risk group, and a low-risk group. Depression and suicidal ideation show a clear, step-wise pattern of differentiation among these subtypes; that is to say, greater overlap of adversity risk is associated with higher levels of depression and suicidal ideation, which is consistent with the cumulative risk theory.

Associations of social support with depression and suicidal ideation vary based on the outcome and configuration of adversity. Parental support and teacher support were both negatively associated with depression. Only teacher support showed an indirect association with suicidal ideation in the single-domain parental-control risk configuration and in overlapping multi-ecological adversity; the parental-support pathway did not reach the corrected significance threshold in either risk group. This pattern does not suggest a lack of family support or absence of teacher support; it is a cross-sectional design, and the number of multiple-adversity groups was small.

Depression and suicidal ideation among college students were related to various ecological disadvantages and differences in perceived social support. A single family-based adversity and parental psychological control were associated with higher depression and suicidal ideation compared to the low-risk group, and overlapping multi-ecological adversity was associated with the highest levels of both outcomes. The association between parental support and lower suicidal ideation appeared to vary according to the circumstances of adversity, while teacher support, as an external resource outside the family, remained significantly associated with suicidal ideation across all profiles; therefore, it is a promising subject for future research on suicide prevention for college students. Therefore, after considering both support pathways, many direct associations remained unexplained; thus, factors other than parental and teacher support may have been responsible.

Together, the present study moves beyond the homogeneity assumption of traditional variable-centered research to identify heterogeneous configurations of multi-ecological adversity among college students in the digital era and shows that parental support and teacher support are differentially associated with depression and suicidal ideation across these configurations, thereby adding to the general knowledge of risk and support for college students’ mental health. Since it is cross-sectional data, only a statistical correlation can be found; causality or intervention effects cannot be determined. However, they can offer some initial support for universities interested in stratified risk screening and family-school collaboration in student mental health work, which future longitudinal and intervention studies need to explore.

The above results of the preliminary study on Chinese university counseling systems show that Chinese colleges have already conducted surveys on the mental health of new students as part of their mental health education (Liu et al., 2017), and this survey can be extended with a few items on parental psychological control, school bullying, and cyberbullying to identify students facing multiple difficulties for follow-up interviews at the counseling center. Counselors and class advisers have regular contact with the students and can provide these students with proactive, autonomy-supportive support; thus, the indirect connection through teacher support for suicidal ideation in adversity profiles remains substantial. Where the primary obstacle is parental control, family-school cooperation can be implemented in the form of parent-education sessions on autonomy-supportive parenting, and parents should not be viewed as the sole source of support. These are only preliminary suggestions and need to be verified by extended observation and intervention studies before implementation.

## Figures and Tables

**Table 1:** Correlation matrix of study variables (*N* = 1142).

Variable	1	2	3	4	5	6	7	8	9	10
1. PPC	1.00									
2. MPC	0.77 ***	1.00								
3. EDV	0.37 ***	0.40 ***	1.00							
4. DPA	0.33 ***	0.34 ***	0.35 ***	1.00						
5. SBV	0.31 ***	0.33 ***	0.50 ***	0.45 ***	1.00					
6. CBV	0.22 ***	0.24 ***	0.39 ***	0.36 ***	0.54 ***	1.00				
7. PS	−0.31 ***	−0.39 ***	−0.25 ***	−0.23 ***	−0.25 ***	−0.20 ***	1.00			
8. TS	−0.28 ***	−0.30 ***	−0.18 ***	−0.29 ***	−0.26 ***	−0.22 ***	0.33 ***	1.00		
9. DEP	0.26 ***	0.31 ***	0.35 ***	0.39 ***	0.48 ***	0.34 ***	−0.30 ***	−0.34 ***	1.00	
10. SI	0.45 ***	0.48 ***	0.42 ***	0.39 ***	0.42 ***	0.36 ***	−0.33 ***	−0.44 ***	0.55 ***	1.00

Note. PPC = paternal psychological control; MPC = maternal psychological control; EDV = exposure to domestic violence; DPA = deviant peer affiliation; SBV = school bullying victimization; CBV = cyberbullying victimization; PS = parental support; TS = teacher support; DEP = depression; SI = suicidal ideation. *** *p* < 0.001.

**Table 2:** Fit indices for latent profile analysis models.

Number of Profiles	Log-Likelihood	AIC	BIC	aBIC	Entropy	BLRT *p*	Profile Proportions
1	−9720	19463	19524	19486	1	-	1.00
2	−8924	17886	17982	17921	0.886	0.002	0.29/0.71
3	−8163	16379	16510	16427	0.924	0.002	0.06/0.28/0.66
4	−8163	16392	16559	16454	0.734	0.325	0.00/0.66/0.28/0.06
5	−7978	16036	16238	16111	0.758	0.002	0.00/0.60/0.28/0.06/0.05
6	−7978	16050	16287	16138	0.592	0.289	0.00/0.00/0.58/0.30/0.05/0.06

Note. AIC = Akaike information criterion; BIC = Bayesian information criterion; aBIC = sample-size-adjusted BIC; BLRT = bootstrap likelihood-ratio test.

**Table 3:** Parallel mediation model for depression.

Contrast	Effect	*B*	β	*SE*	*p*	Bootstrap CI
Class 1 vs. Class 3	Direct effect	0.647	0.273	0.096	<0.001	[0.461, 0.845]
Class 1 vs. Class 3	Indirect via parental support	0.082	0.035	0.025	0.001	[0.026, 0.154]
Class 1 vs. Class 3	Indirect via teacher support	0.102	0.043	0.020	<0.001	[0.057, 0.159]
Class 1 vs. Class 3	Total effect	0.831	0.350	0.092	<0.001	[0.651, 1.014]
Class 2 vs. Class 3	Direct effect	0.133	0.106	0.039	0.001	[0.058, 0.209]
Class 2 vs. Class 3	Indirect via parental support	0.057	0.045	0.017	0.001	[0.019, 0.103]
Class 2 vs. Class 3	Indirect via teacher support	0.076	0.060	0.013	<0.001	[0.044, 0.109]
Class 2 vs. Class 3	Total effect	0.266	0.211	0.035	<0.001	[0.198, 0.336]

Note. Class 1 = multiple-adversity high-risk group; Class 2 = parental-control risk group; Class 3 = low-risk group. The low-risk group served as the control. Bonferroni-adjusted inference for indirect effects was conducted using the 98.75% percentile bootstrap confidence interval (CI), and the direct and total effects were reported with 95% confidence intervals.

**Table 4:** Parallel mediation model for suicidal ideation.

Contrast	Effect	*B*	β	*SE*	*p*	Bootstrap CI
Class 1 vs. Class 3	Direct effect	0.897	0.338	0.078	<0.001	[0.742, 1.041]
Class 1 vs. Class 3	Indirect via parental support	0.054	0.020	0.026	0.039	[−0.011, 0.127]
Class 1 vs. Class 3	Indirect via teacher support	0.150	0.056	0.027	<0.001	[0.089, 0.224]
Class 1 vs. Class 3	Total effect	1.100	0.414	0.075	<0.001	[0.952, 1.241]
Class 2 vs. Class 3	Direct effect	0.364	0.259	0.047	<0.001	[0.268, 0.457]
Class 2 vs. Class 3	Indirect via parental support	0.037	0.027	0.018	0.036	[−0.007, 0.082]
Class 2 vs. Class 3	Indirect via teacher support	0.112	0.080	0.017	<0.001	[0.069, 0.157]
Class 2 vs. Class 3	Total effect	0.513	0.365	0.041	<0.001	[0.431, 0.592]

Note. Class 1 = multiple-adversity high-risk group; Class 2 = parental-control risk group; Class 3 = low-risk group. The Low-Risk Group served as the reference. Bonferroni-adjusted inference for indirect effects used the 98.75% percentile bootstrap confidence interval (CI), and the direct and total effects were shown with 95% confidence intervals.

## Data Availability

The original contributions presented in this study are included in the article. Further inquiries can be directed to the corresponding authors.

## Supplementary Materials

The following supporting information can be downloaded at: https://www.mdpi.com/article/10.3390/bs16101934/s1. Table S1: Average posterior class-membership probabilities for the three-profile solution; Table S2: Covariate-adjusted omnibus tests and adjusted means with 95% confidence intervals for depression (PHQ-9), suicidal ideation and depression (PHQ-8) across adversity profiles; Table S3: Tukey-corrected pairwise comparisons of covariate-adjusted outcomes across adversity profiles; Table S4: Parallel indirect-association model for depression using PHQ-8 (sensitivity analysis); Table S5: Two-profile solution: profile sizes, entropy and mean assigned posterior probabilities; Table S6: Covariate-adjusted comparisons of PHQ-9, suicidal ideation and PHQ-8 between the two profiles; Table S7: Direct and total associations between two-profile membership and PHQ-9, suicidal ideation and PHQ-8.
