# Supplementary material for: Social Support Across Adversity Subtypes: A Latent Profile Analysis of Depression and Suicidal Ideation in College Students
Source: Behav Sci (Basel). 2026 Oct 17;16(10):1934. doi: 10.3390/bs16101934 (PMC13701571; doi:10.3390/bs16101934)
Supplement: Supplementary file 1 [file behavsci-16-01934-s001.zip › behavsci-4571420-supplementary.pdf]

Supplementary Materials

Table S1. Average posterior class-membership probabilities for the three-profile solution.

Class	Label	n	P(Class 1: High risk)	P(Class 2: Parental control)	P(Class 3: Low risk)
1	high risk	68	**0.985**	0.014	0.000
2	parental control	316	0.003	**0.942**	0.055
3	low risk	758	0.001	0.026	**0.974**

Note. Posterior probabilities are averaged within the assigned class. Four- to six-profile solutions contained an empty or effectively empty class.

Table S2. Covariate-adjusted omnibus tests and adjusted means with 95% confidence intervals for depres-sion (PHQ-9), suicidal ideation and depression (PHQ-8) across adversity profiles.

Outcome	Overall profile effect	p	η^2_p
PHQ9_mean	F(2, 1137) = 96.68	<0.001	0.145
suicidal_ideation_mean	F(2, 1137) = 198.14	<0.001	0.258
PHQ8_mean	F(2, 1137) = 84.20	<0.001	0.129
**Outcome**	**Profile**	**Adjusted mean**	**95% CI**
	low risk	1.434	[1.397, 1.471]
PHQ9_mean	parental control	1.692	[1.634, 1.749]
	high risk	2.256	[2.132, 2.380]
	low risk	1.567	[1.528, 1.605]
suicidal_ideation_mean	parental control	2.076	[2.016, 2.136]
	high risk	2.662	[2.532, 2.792]
	low risk	1.472	[1.433, 1.511]
PHQ8_mean	parental control	1.729	[1.669, 1.790]
	high risk	2.276	[2.145, 2.407]

Note. Models adjust for sex and centered age. All Tukey-adjusted pairwise profile comparisons were $p < 0.001$.

Table S3. Tukey-corrected pairwise comparisons of covariate-adjusted outcomes across adversity profiles.

Outcome	Contrast	Effect	*B*	*β*	*SE*	*p*	bootstrap CI
PHQ-9	Class 1 vs. Class 3	Direct effect	0.641	0.270	0.095	< 0.001	[0.456, 0.821]
	Class 1 vs. Class 3	Indirect via parental support	0.079	0.033	0.025	0.002	[0.023, 0.150]
	Class 1 vs. Class 3	Indirect via teacher support	0.102	0.043	0.021	< 0.001	[0.055, 0.161]
	Class 1 vs. Class 3	Total effect	0.822	0.346	0.093	< 0.001	[0.640, 1.000]
	Class 2 vs. Class 3	Direct effect	0.128	0.102	0.040	0.001	[0.051, 0.208]
	Class 2 vs. Class 3	Indirect via parental support	0.055	0.043	0.017	0.001	[0.015, 0.098]
	Class 2 vs. Class 3	Indirect via teacher support	0.076	0.060	0.014	< 0.001	[0.046, 0.115]
	Class 2 vs. Class 3	Total effect	0.258	0.206	0.035	< 0.001	[0.190, 0.327]
Suicidal ideation	Class 1 vs. Class 3	Direct effect	0.891	0.336	0.080	< 0.001	[0.735, 1.046]
	Class 1 vs. Class 3	Indirect via parental support	0.054	0.020	0.027	0.048	[-0.013, 0.124]
	Class 1 vs. Class 3	Indirect via teacher support	0.150	0.057	0.027	< 0.001	[0.086, 0.227]
	Class 1 vs. Class 3	Total effect	1.095	0.413	0.078	< 0.001	[0.942, 1.244]
	Class 2 vs. Class 3	Direct effect	0.361	0.257	0.047	< 0.001	[0.273, 0.455]
	Class 2 vs. Class 3	Indirect via parental support	0.037	0.026	0.018	0.042	[-0.009, 0.083]
	Class 2 vs. Class 3	Indirect via teacher support	0.111	0.079	0.017	< 0.001	[0.074, 0.161]
	Class 2 vs. Class 3	Total effect	0.510	0.363	0.041	< 0.001	[0.429, 0.593]
PHQ-8	Class 1 vs. Class 3	Direct effect	0.612	0.247	0.097	< 0.001	[0.420, 0.795]
	Class 1 vs. Class 3	Indirect via parental support	0.086	0.035	0.027	0.001	[0.026, 0.159]
	Class 1 vs. Class 3	Indirect via teacher support	0.106	0.043	0.022	< 0.001	[0.057, 0.170]
	Class 1 vs. Class 3	Total effect	0.804	0.324	0.094	< 0.001	[0.621, 0.984]
	Class 2 vs. Class 3	Direct effect	0.119	0.091	0.042	0.004	[0.041, 0.202]
	Class 2 vs. Class 3	Indirect via parental support	0.059	0.045	0.017	0.001	[0.017, 0.104]
	Class 2 vs. Class 3	Indirect via teacher support	0.079	0.060	0.014	< 0.001	[0.048, 0.121]
	Class 2 vs. Class 3	Total effect	0.257	0.196	0.037	< 0.001	[0.187, 0.327]

Note. Class 1 = multiple-adversity high-risk group; Class 2 = parental-control risk group; Class 3 = low-risk group. The low-risk group was the reference category. Estimates were based on 2,000 bootstrap samples. Specific indirect effects are reported with 98.75% percentile bootstrap confidence intervals; direct and total effects are reported with 95% confidence intervals.

Table S4. Parallel indirect-association model for depression using PHQ-8 (sensitivity analysis).

Depression score	Outcome	n	r	95% CI	p
PHQ-9	Suicidal ideation	1142	0.548	[0.506, 0.587]	<0.001
PHQ-8	Suicidal ideation	1142	0.516	[0.472, 0.557]	<0.001

The adjusted profile effect for PHQ-8 was $F(2, 1137) = 84.20$, $p < 0.001$, $\eta^2 p = 0.129$. The PHQ-8 indirect-association estimates are included in Table S3.

Table S5. Two-profile solution: profile sizes, entropy and mean assigned posterior probabilities.

Profile	n	%	Mean assigned posterior	FPC	MPC	FVE	DPA	SBV	CBV
Lower risk	809	70.8	0.977	-0.476	-0.530	-0.283	-0.231	-0.242	-0.174
Higher risk	333	29.2	0.945	1.156	1.286	0.687	0.560	0.588	0.423

Note. Values for FPC, MPC, FVE, DPA, SBV, and CBV are standardized indicator means. The two-profile solution was estimated with Model 1 (equal variances and zero covariances): log-likelihood = -8923.95, AIC = 17885.91, BIC = 17981.68, sample-size-adjusted BIC = 17921.33, and entropy = 0.886. FPC = father psychological control; MPC = mother psychological control; FVE = family violence exposure; DPA = deviant peer affiliation; SBV = school bullying victimization; CBV = cyberbullying victimization.

Table S6. Covariate-adjusted comparisons of PHQ-9, suicidal ideation and PHQ-8 between the two profiles.

Outcome	Lower risk, adjusted M (95% CI)	Higher risk, adjusted M (95% CI)	Difference (95% CI)	F(1, 1138)	p	Partial eta squared
Depressive symptoms (PHQ-9)	1.451 (1.414, 1.488)	1.804 (1.745, 1.862)	0.353 (0.283, 0.422)	107.14	<.001	0.086
Suicidal ideation	1.590 (1.551, 1.628)	2.218 (2.157, 2.278)	0.628 (0.556, 0.700)	301.74	<.001	0.210
Depressive symptoms (PHQ-8)	1.490 (1.451, 1.529)	1.836 (1.774, 1.897)	0.346 (0.273, 0.418)	93.89	<.001	0.076

Note. Models adjusted for sex and centered age. The difference is the adjusted higher-risk minus lower-risk contrast. With two profiles, this is the only pairwise comparison; its 95% confidence interval is equivalent to the Tukey-adjusted interval.

Table S7. Direct and total associations between two-profile membership and PHQ-9, suicidal ideation and PHQ-8.

Outcome	Contrast	Effect	*B*	*β*	*SE*	*p*	bootstrap CI
PHQ-9	Higher vs. lower risk	Direct effect	0.203	0.164	0.043	< 0.001	[0.120, 0.285]
	Higher vs. lower risk	Indirect via parental support	0.070	0.057	0.019	< 0.001	[0.026, 0.121]
	Higher vs. lower risk	Indirect via teacher support	0.080	0.065	0.014	< 0.001	[0.050, 0.120]
	Higher vs. lower risk	Total effect	0.353	0.285	0.038	< 0.001	[0.279, 0.427]
Suicidal ideation	Higher vs. lower risk	Direct effect	0.459	0.332	0.048	< 0.001	[0.369, 0.556]
	Higher vs. lower risk	Indirect via parental support	0.051	0.037	0.020	0.011	[-0.0004, 0.103]
	Higher vs. lower risk	Indirect via teacher support	0.117	0.085	0.018	< 0.001	[0.078, 0.164]
	Higher vs. lower risk	Total effect	0.628	0.454	0.042	< 0.001	[0.551, 0.715]
PHQ-8	Higher vs. lower risk	Direct effect	0.187	0.145	0.044	< 0.001	[0.103, 0.272]
	Higher vs. lower risk	Indirect via parental support	0.075	0.058	0.020	< 0.001	[0.030, 0.128]
	Higher vs. lower risk	Indirect via teacher support	0.083	0.065	0.015	< 0.001	[0.053, 0.125]
	Higher vs. lower risk	Total effect	0.346	0.268	0.040	< 0.001	[0.269, 0.423]

Note. Models adjusted for sex and centered age. Estimates were based on 2,000 bootstrap samples; all 2,000 samples were successful for each outcome. The higher-risk profile was coded 1 and the lower-risk profile 0. Specific indirect effects are reported with 98.75th percentile bootstrap confidence intervals; direct and total effects are reported with 95% confidence intervals.
